# Characterization of the Relationship between APOBEC3B Deletion and ACE Alu Insertion

**DOI:** 10.1371/journal.pone.0064809

**Published:** 2013-05-24

**Authors:** Kang Wang, Yuanyuan Li, Chunyan Dai, Kaishi Wang, Jinghua Yu, Yiran Tan, Wenyan Zhang, Xiao-Fang Yu

**Affiliations:** 1 Institute of Virology and AIDS Research, First Hospital of Jilin University, Changchun, Jilin, People's Republic of China; 2 Department of Molecular Microbiology and Immunology, Johns Hopkins Bloomberg School of Public Health, Baltimore, Maryland, United States of America; Tel Aviv University, Israel

## Abstract

The insertion/deletion (I/D) polymorphism of the angiotensin converting enzyme (ACE), commonly associated with many diseases, is believed to have affected human adaptation to environmental changes during the out-of-Africa expansion. APOBEC3B (A3B), a member of the cytidine deaminase family APOBEC3s, also exhibits a variable gene insertion/deletion polymorphism across world populations. Using data available from published reports, we examined the global geographic distribution of ACE and A3B genotypes. In tracking the modern human dispersal routes of these two genes, we found that the variation trends of the two I/D polymorphisms were directly correlated. We observed that the frequencies of ACE insertion and A3B deletion rose in parallel along the expansion route. To investigate the presence of a correlation between the two polymorphisms and the effect of their interaction on human health, we analyzed 1199 unrelated Chinese adults to determine their genotypes and other important clinical characteristics. We discovered a significant difference between the ACE genotype/allele distribution in the A3B DD and A3B II/ID groups (P = 0.045 and 0.015, respectively), indicating that the ACE Alu I allele frequency in the former group was higher than in the latter group. No specific clinical phenotype could be associated with the interaction between the ACE and A3B I/D polymorphisms. A3B has been identified as a powerful inhibitor of Alu retrotransposition, and primate A3 genes have undergone strong positive selection (and expansion) for restricting the mobility of endogenous retrotransposons during evolution. Based on these findings, we suggest that the ACE Alu insertion was enabled (facilitated) by the A3B deletion and that functional loss of A3B provided an opportunity for enhanced human adaptability and survival in response to the environmental and climate challenges arising during the migration from Africa.

## Introduction

Insertion/deletion (I/D) polymorphism of the angiotensin converting enzyme (ACE) gene is an important genetic marker that has been used in numerous studies [1]–[8]. ACE is a key enzyme of the renin-angiotensin system (RAS) and is widely distributed in human tissues, including the vascular endothelium, intestinal epithelium, kidney, lung, and testes [9], [10]. The enzyme plays a vital role in the regulation of systemic blood pressure and renal electrolyte homeostasis by converting the inactive angiotensin I to the vasoconstrictor angiotensin II and inactivating the proinflammatory vasodilator bradykinin [9]. In *Homo sapiens*, the gene encoding ACE is located on the long arm of chromosome 17 and comprises 26 exons and 25 introns [11]. The insertion/deletion polymorphism is characterized by the presence (insertion) or absence (deletion) of a 287-base pair (bp) Alu repeat within intron 16, producing three genotypes: II, DD homozygote, and ID heterozygote [1]. The deletion allele was found to be associated with higher plasma ACE concentrations; that is, the average plasma ACE level of persons with the various genotypes follows the order DD>ID>II [1]. The I/D genotype distribution of ACE shows apparent variations among different ethnic populations throughout the world [8]. A number of research papers have reported a significant association between ACE I/D polymorphism and a series of diseases, including hypertension, type 2 diabetes, cardiovascular disease, kidney disease, cancer, and obesity [2]–[7].

APOBEC3B (A3B) belongs to the apolipoprotein B mRNA editing catalytic polypeptide-like 3 (APOBEC3) protein family, whose members all possess cytidine deaminase activity and are therefore able to convert cytosine to uracil [12], [13]. Most A3 proteins can restrict the replication of retroviruses or retrotransposition of endogenous elements to varying degrees [13]–[18]. A3B, in particular, has been shown to inhibit replication of HBV and HIV as well as the retrotransposition of Line1 and Alu [13], [15], [16], [17], [19]. The human A3B gene is located on the long arm of chromosome 22, clustered with other A3 genes [12]. The gene is widely expressed at a low level in PBMCs, colon, lung, spleen, liver, ovary, and testis [20]. A common 29.5-kb gene deletion polymorphism that removes the entire A3B coding region has been identified and, like the ACE I/D polymorphism, its distribution varies significantly in diverse ethnic groups [21]. A3B I/D polymorphism has been suggested to influence host susceptibility to HBV infection and falciparum malaria [22], [23].

The APOBEC3 gene family has undergone expansion during species evolution, increasing from a single copy in rodents to at least seven copies in primates [24]. It is believed that the APOBEC3 family has been subjected to strong and continuous selective pressure throughout primate evolution [25], [26]. The A3B genotype distribution in world populations indicates a weak selection for the deletion [21]. In addition, the ACE D allele also presents a striking geographic distribution, showing evidence of “signatures of selection” that follows the out-of-Africa route [8].

The formation of A3B I/D polymorphism was caused by A3B deletion, while the formation of ACE I/D polymorphism was caused by the ACE Alu insertion. To investigate whether a correlation exists between the two I/D polymorphisms or between the A3B deletion and the ACE Alu insertion, we compared the genotype distributions of ACE and A3B across the world. The result of our analysis demonstrates that their change trends are almost completely opposite to each other along the modern human dispersal route. Based on this interesting discovery, we investigated their distributions and interaction effects on the clinical characteristics of a random sample of Chinese population. The combination of the ACE and A3B genotypes/alleles in the study population was not completely stochastic, but no interaction effects were embodied in the clinical characteristics.

## Materials and Methods

### Ethics Statement

The study protocol for the random population was approved by the ethics committee at the First Hospital of Jilin University. All participants or their parents gave written informed consent.

### Publication selection and data extraction

A search was performed in the PubMed database to identify published studies reporting A3B or ACE genotype/allele distributions. The retrieved studies were manually screened to assess their appropriateness for inclusion. A3B genotype distributions in world populations have mainly been investigated and analyzed thus far by Kidd et al. [21]. A3B allele frequencies in various geographic regions were extracted and cited directly from the supplemental materials of this paper, except for Middle South Asia, because its data actually only included Pakistanis and few Uygur. The A3B deletion distribution in India has been reported in another study characterizing 25 ethnic populations [23]. Therefore, we extracted data from both studies to evaluate A3B deletion frequency in Middle South Asia in particular. Li et al. have compiled a worldwide spatial database on ACE I/D frequency distributions using data derived from 299 published sources, including a total of 183,555 individuals from 422 sampling populations [8]. ACE genotype/allele distributions of some nations were extracted from the database to calculate ACE allele frequencies in Africa, Middle East, Europe, Middle South Asia, and East Asia, respectively, supplemented with related data for Pakistanis and Khoisan from the two original studies [27], [28]. ACE genotype/allele distributions among the aboriginals of America and Australia were collected from several other papers [28]–[32].

We also extracted the data of the frequencies of other three Alu insertions (called TPA 25, PV92 and FXIIIB, respectively) among different populations from one study to calculate their geographic distributions [28].

### Study Population

The subjects were selected by a simple random sampling approach from persons who visited the general health check-up center of the First Hospital of Jilin University in 2010. Subjects who were pregnant or were suffering from serious illnesses were excluded from the investigation to ensure the validity of results. A group of 1199 adults (683 males, 516 females) from the participants were genotyped for ACE and A3B I/D polymorphisms, in addition to being measured for some specific clinical characteristics according to their individual willingness to undergo testing. Another 1581 participants (756 males, 825 females) were also randomly chosen to supplement the number of A3B deletion homozygotes. These persons were initially genotyped for A3B polymorphism, and only those identified as A3B deletion homozygotes were then genotyped for ACE polymorphism. All participants were unrelated Han Chinese and local residents of Jilin Province in northeast China.

### Measurement of clinical characteristics

All measurements were performed by skilled technicians in the early morning after participants had fasted overnight. Body weight and height were measured twice in light indoor clothing without shoes, and body mass index (BMI) was calculated as body weight divided by the square of height (kg/m^2^). Heart rate was determined from the resting electrocardiogram recorded by full automatic electrocardiograph (Nihon Kohden ECG-9130P, Tokyo, Japan). Blood pressure was measured at least twice with an electronic sphygmomanometer (Omron BP-203RVIIIC, Kyoto, Japan) from the right arm of subjects in the seated position after 5 minutes of rest according to a standard protocol. These anthropometric and physiological parameters were measured at the general health check-up center of the First Hospital of Jilin University.

Fasting blood samples were drawn from the antecubital vein by nurses, while urine samples were collected by participants themselves according to the instructions of nurses. Blood and urine biochemistry were measured in the Department of Laboratory Medicine, First Hospital of Jilin University. The concentrations of serum lipid, plasma glucose, blood urea nitrogen and hepatic function indexes were assayed by standard enzymatic methods in fresh blood samples using commercial reagent kits (Kehua Bioengineering, Shanghai, China) with an autoanalyzer (Hitachi 7600, Tokyo, Japan). Urinary protein and occult blood were tested in fresh urine samples using urinalysis strips with an automated urine analyzer (DIRUI H-800, Changchun, China). Urinary protein test results were categorized based on semi-quantitative values as: (―); (1+) for 0.3 g/l; (2+) for 1.0 g/l; (3+) for 3.0 g/L. Urine occult blood results were categorized based on semi-quantitative values as: (―); (±) for 10 erythrocytes/µl; (1+) for 25 erythrocytes/µl; (2+) for 80 erythrocytes/µl; (3+) for 200 erythrocytes/µl.

### Genotyping

Genomic DNA was extracted from EDTA blood with Blood DNA Mini Kits (SIMGEN Biotech, Hangzhou, China). Genotyping was performed on the extracted DNA by polymerase chain reaction (PCR) with specific oligonucleotide primers.

Genotypes of ACE I/D polymorphism were detected according to the methods of Rigat et al. and Lindpaintner et al. [33], [34], [35]. To guarantee the reliability of the genotyping results, all samples were amplified with both primer pairs (5′-CTGGAGA CCACTCCCATCCTTTCT-3′ and 5′-GATGTGGCCATCACATTCGTCAGAT-3′; 5′-TGGGACCACAGCGCCCGCCACTAC-3′ and 5′-TCGCCAGCCCTCCCATGC CCATAA-3′) simultaneously. The first pair of primers was expected to amplify a 490-bp DNA fragment for the insertion allele or a 190-bp DNA fragment for the deletion allele, while the second pair of primers was designed to amplify a 335-bp DNA fragment only in the presence of the insertion allele.

A3B I/D polymorphism was genotyped with PCR primers designed by Kidd et al. [21]. One pair of primers (5′-TAGGTGCCACCCCGAT-3′ and 5′-TTGAGC ATAATCTTACTCTTGTAC-3′) was used to identify the presence of the deletion allele, and two pairs of primers (Insertion 1: 5′-TTGGTGCTGCCCCCTC-3′ and 5′-TAGAGACTGAGGCCCAT-3′; Insertion 2: 5′-TGTCCCTTTTCAGAGTTTGA GTA-3′ and 5′-TGGAGCCAATTAATCACTTCAT-3′) were used to identify the presence of the insertion allele. Insertion 1 primers were only applied to those samples showing negative amplification results with the Insertion 2 primers (1199 adults), while both primer pairs were used for genotyping the other 1581 persons. PCR experiments were repeated at least twice for every sample to ensure the accuracy of the genotyping results.

PCR reactions were performed on a MyCycler™ Thermal Cycler (BIO-RAD, Hercules, USA) with a high-fidelity Taq polymerase kit (TransGen Biotech, Beijing, China) according to the manufacturer's protocol and set for 30 cycles with the same temperature parameters as described previously [21], [33], [34], [35]. PCR products were separated by electrophoresis on a 1.5% agarose gel mixed with ethidium bromide and visualized under ultraviolet light to judge the genotype.

### Data analysis

ACE allele frequency in a geographic region was calculated as the average value for several representative countries (File S1). If there was more than one published source reporting the ACE genotype/allele distribution of the same country, then several main publications were selected together to calculate the frequency in the country by the gene-counting method. Two African ethnic groups, Pygmy and Khoisan, were included together with the relevant countries in order to calculate the ACE insertion frequency in Africa. To evaluate the A3B deletion frequency in Middle South Asia, the deletion frequency among Indians was first computed as the mean value for 25 ethnic populations. Then, the Indian deletion value and the deletion value in Middle South Asia reported by Kidd et al. (mainly as Pakistani) were added together and averaged again to give a final result (File S1). The frequencies of three additional Alu insertions in a geographic region were calculated as the average value for several representative populations (File S1).

The chi-square test was used to assess whether A3B and ACE genotype distributions in the study population were in Hardy-Weinberg equilibrium (HWE) and to compare ACE allele/genotype distributions in different A3B genotype groups. To explore the possible effects of the interaction of the ACE and A3B I/D polymorphisms on clinical characteristics, the 1199 adults were grouped into 1) carriers of both D alleles and others, and 2) carriers of both the ACE D and A3B I alleles and others. For a control, we also compared the characteristics of different ACE genotype groups. The Kolmogorov-Smirnov test was used to examine the normality of continuous variables, and any skewed data were log-transformed in order to normalize their distributions. Normalized data were compared between two groups by unpaired Student's t-test and among three groups by one-way analysis of variance (ANOVA). Skewed data that could not be normalized, as well as categorical variables, were compared by nonparametric Mann-Whitney U test. If the P-value was <0.1, analysis of covariance (ANCOVA) was employed to include age and BMI as covariates to adjust the P-value. All statistical analyses were performed with SPSS Version 19.0 software (SPSS Inc., Chicago, USA). A two-tailed P-value of <0.05 was considered statistically significant.

## Results

### Comparison of the global distributions of ACE and A3B I/D polymorphisms

Li et al. have discovered that ACE deletion shows an obvious decreasing geographic genetic cline following the route of out-of-Africa expansion from East Africa [8]. In the report of Kidd et al., A3B deletion was shown to be almost null in Africans, rare in Middle East populations and Europeans, slightly more frequent in Central South Asians, common in East Asians and American Indians, and almost fixed in Oceanic populations [21]. The A3B deletion also displayed an evident increasing trend following the out-of-Africa expansion route in the 51 different ethnic populations. Taken together, these data indicate that ACE and A3B I/D polymorphisms show opposite geographic distributions along the modern human dispersal route.

**Figure 1:**
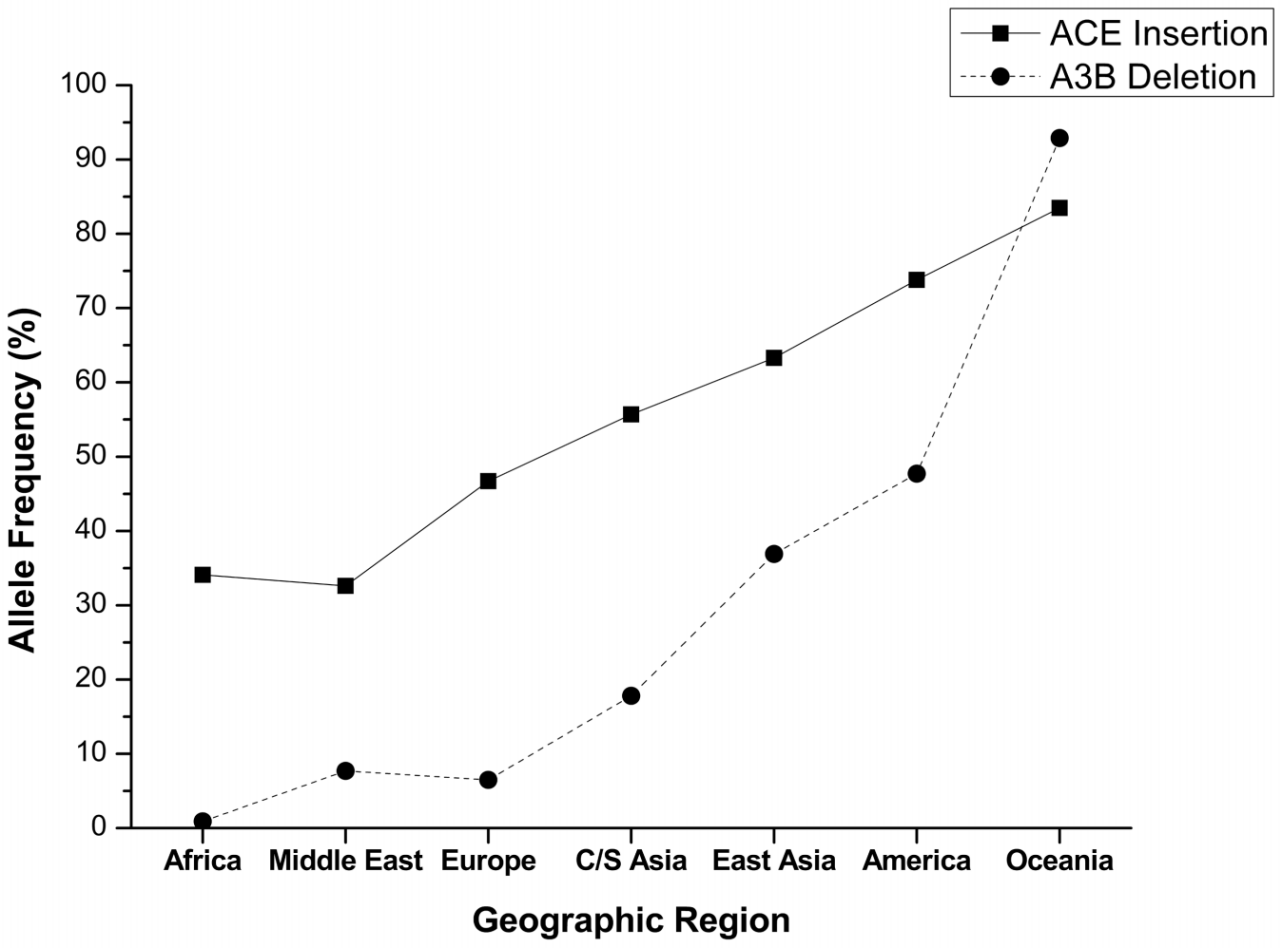
Variation curves of ACE insertion and A3B deletion distributions among different geographic regions. ‘C/S Asia’ corresponds to Central South Asia. America refers to Amerindians; Oceania refers to Oceania aboriginals.

Because the sample sizes of some ethnic populations in the report of Kidd et al. were too small to guarantee the precision of the A3B allele/genotype frequencies estimated, we chose to compare the frequencies of the ACE I and A3B D alleles in different geographic regions to give a concrete and intuitional impression. ACE insertion frequencies were calculated to be 34.1, 32.6, 46.7, 55.7, 63.3, 73.8, and 83.5 percent in Africa, the Middle East, Europe, Central South Asia, East Asia, America, and Oceania, respectively (Table S1). A3B deletion frequencies were cited or calculated to be 0.9, 7.7, 6.5, 17.8, 36.9, 47.7 and 92.9 percent, respectively, in those same geographic regions (Table S1). The results clearly confirmed the opposite variation trends of ACE and A3B I/D polymorphisms along the human dispersal route. Variation curves for ACE insertion and A3B deletion among the geographic regions were plotted based on the results. Amazingly, their variation curves were observed to be almost parallel, both rising continuously along the out-of-Africa expansion route (Fig. 1).

The distributions of three additional Alu insertions in different geographic regions presented variation trends similar to that of the ACE Alu insertion (Table S1).

### ACE and A3B genotype/allele distributions in the study population

The 1199 adults in the study were aged 41.6±12.3 years (mean ± SD), with a range of 18 to 84 years. The average age of the other 1581 subjects was 42.5±14.5 years, with a range of 15 to 86 years. Most of the participants were genotyped successfully and accurately, with the exception of three whose DNA failed to amplify. No contradictory ACE genotyping results were found for the PCR of the two pairs of primers.

The ACE genotype/allele distribution in the 1199 adults and A3B genotype/allele distribution in all participants are listed in Table 1. The frequencies of the ACE I and D alleles were 0.674 and 0.326, respectively, consistent with previous reports (File S1). The genotype distribution of ACE was almost completely identical to the expected Hardy-Weinberg frequency (P = 0.962). The A3B genotype/allele frequencies did not differ between the 1199 adults and the other 1581 subjects or between males and females (Table 1, Table S2). The A3B D allele frequency was 0.355, which is very close to the frequency among the Han Chinese population and East Asians reported by Kidd et al. [21]. Even so, the A3B genotype distribution deviated significantly from HWE (P<0.001). Thus, we did not pursue the analysis of the A3B genotype/allele frequencies or values for clinical characteristics in the different A3B genotype groups in this study.

**Table 1:** A3B and ACE genotype/allele distributions in the study population.

		Genotype counts (HWE expected)			Allele frequency		
Gene	Population	II	ID	DD	I	D	HWE P-value
ACE	1199 adults	542(545)	533(527)	124(127)	0.674	0.326	0.962
A3B	1199 adults	470 (514)	630 (542)	99 (143)	0.655	0.345	<0.001
A3B	1581 subjects	624 (676)	818 (714)	136 (188)	0.655	0.345	<0.001
A3B	Total	1094 (1190)	1448 (1256)	235 (331)	0.655	0.345	<0.001

ACE genotype/allele distributions in different A3B genotype groups were compared, and significant differences were discovered between the A3B DD group and the II/ID group (P = 0.045 and 0.015, respectively; Table 2). The distribution of the ACE allele in the A3B DD group of 1199 adults was approximately the same as that of the other 1581 subjects. The frequency of the ACE D allele in the A3B DD group (0.272) was lower than that in the A3B II/ID group (0.33). The ACE ID and DD genotype frequencies in the A3B DD group (0.417 and 0.064, respectively) were both lower than in the A3B II/ID group (0.444 and 0.108, respectively). No significant difference was found in the ACE genotype/allele distribution of the A3B II and A3B ID groups (data not shown).

**Table 2:** ACE genotype/allele distributions in different A3B genotype groups.

A3B genotype group	ACE genotype counts (frequencies)	Added up	P-value	Allele counts (frequencies)	P-value
II in 1199 adults	II = 222 (0.472)				
	ID = 199 (0.423)				
	DD = 49 (0.105)	II = 493 (0.448)		I = 1474 (0.67)	
ID in 1199 adults	II = 271 (0.430)	ID = 488 (0.444)		D = 726 (0.33)	
	ID = 289 (0.459)	DD = 119 (0.108)			
	DD = 70 (0.111)				
DD in 1199 adults	II = 49 (0.495)		0.045		0.015
	ID = 45 (0.454)				
	DD = 5 (0.051)	II = 122 (0.519)		I = 342 (0.728)	
DD in 1581 subjects	II = 73 (0.537)	ID = 98 (0.417)		D = 128 (0.272)	
	ID = 53 (0.390)	DD = 15 (0.064)			
	DD = 10 (0.073)				

### Interaction effects between ACE and A3B I/D polymorphisms on clinical characteristics

No significant differences were found between the clinical characteristics of the carriers of both the ACE D and A3B I alleles and the remainder among the males or females of the 1199 adults, although there was a strong trend for aspartate aminotransferase in females (P = 0.051; Table 3 and Table 4). The same results were obtained when the subjects were grouped into ACE/A3B D allele carriers and others (Table S3 and Table S4). As a control, the ACE genotype itself was only observed to be significantly related to blood urea nitrogen values among the male subjects (P = 0.031). This is a finding that, to our knowledge, has not been previously reported (Table S5 and Table S6).

**Table 3:** Characteristics of male subjects grouped by ACE and A3B genotypes.

	Mean ± SD (No. of subjects measured)		
Characteristic	ACE D/A3B I carriers	others	P-value
Age (years)	41.1±11.9 (340)	41.5±12.4 (343)	0.692
BMI (kg/m^2^)	26.2±3.2 (296)	25.8±3.3 (297)	0.220
Heart rate (beats/min)	76.7±10.6 (305)	76.3±10.7 (305)	0.637
Blood pressure (mm Hg)			
Systolic	135.9±18.6 (305)	133.7±17.9 (305)	0.201
Diastolic	83.8±11.9 (305)	83.1±11.4 (305)	0.490
Plasma glucose (mmol/L)	5.81±1.91 (304)	5.58±1.14 (312)	0.629
Serum lipid (mmol/L)			
Total cholesterol	4.92±0.87 (304)	4.83±0.92 (313)	0.153
Triglycerides	1.94±1.41 (304)	1.80±1.36 (313)	0.098
HDL-cholesterol	1.54±0.54 (253)	1.55±0.57 (261)	0.943
LDL-cholesterol	3.11±0.81 (253)	3.05±0.76 (261)	0.513
HDL-C/LDL-C ratio	0.52±0.21 (253)	0.53±0.23 (261)	0.701
Renal function indexes			
BUN (mmol/L)	5.27±1.29 (305)	5.40±1.35 (314)	0.222
Urinary protein	——a (319)	——a (328)	0.956
Urinary occult blood	——a (319)	——a (328)	0.155
Liver function indexes (U/L)			
ALT	32.9±25.0 (323)	31.0±19.2 (332)	0.915
γ–GT	45.2±45.6 (323)	43.5±43.4 (332)	0.735
AST	26.0±12.4 (323)	24.8±9.3 (333)	0.784
Triglycerides	1.95±1.45 (284)	1.77±1.36 (291)	0.103^b^

a belong to categorical variables.

b p-value from analysis of covariance including subjects whose BMI data were available.

Abbreviations: BMI, body mass index; HDL, high density lipoprotein; LDL, low density lipoprotein; BUN, blood urea nitrogen; ALT, alanine aminotransferase; γ–GT, gamma-glutamyl transpeptidase; AST, aspartate aminotransferas.

**Table 4:** Characteristics of female subjects grouped by ACE and A3B genotypes.

	Mean ± SD (No. of subjects measured)		
Characteristic	ACE D/A3B I carriers	others	P-value
Age (years)	42.2±12.5 (267)	41.7±12.5 (249)	0.740
BMI (kg/m^2^)	23.5±3.4 (231)	23.4±3.2 (217)	0.965
Heart rate (beats/min)	80.2±11.7 (245)	79.5±10.7 (228)	0.718
Blood pressure (mm Hg)			
Systolic	125.9±19.9 (245)	125.9±19.5 (228)	0.995
Diastolic	75.8±11.4 (245)	76.0±12.7 (228)	0.962
Plasma glucose (mmol/L)	5.24±0.72 (241)	5.27±0.82 (230)	0.431
Serum lipid (mmol/L)			
Total cholesterol	4.80±0.90 (241)	4.80±0.91 (226)	0.825
Triglycerides	1.26±0.86 (241)	1.25±0.91 (226)	0.990
HDL-cholesterol	1.88±0.70 (191)	2.00±0.75 (186)	0.134
LDL-cholesterol	2.90±0.77 (191)	2.85±0.76 (186)	0.522
HDL-C/LDL-C ratio	0.71±0.36 (191)	0.76±0.40 (186)	0.181
Renal function indexes			
BUN (mmol/L)	4.62±1.29 (239)	4.53±1.09 (227)	0.784
Urinary protein	——a (236)	——a (232)	0.408
Urinary occult blood	——a (236)	——a (232)	0.474
Liver function indexes (U/L)			
ALT	22.0±35.3 (248)	19.1±15.5 (238)	0.214
γ–GT	21.4±19.1 (248)	21.8±19.5 (238)	0.650
AST	22.8±17.1 (248)	20.8±9.7 (240)	0.051^b^

a belong to categorical variables.

b Analysis of covariance can not be employed because the data can't be transformed to normality.

Abbreviations: BMI, body mass index; HDL, high density lipoprotein; LDL, low density lipoprotein; BUN, blood urea nitrogen; ALT, alanine aminotransferase; γ–GT, gamma-glutamyl transpeptidase; AST, aspartate aminotransferase.

## Discussion

In the present study, we have observed that the ACE Alu insertion genotype is not randomly distributed among individuals with different A3B deletion genotypes. People who are homozygous for A3B deletions (A3B DD) have higher rate of ACE Alu insertion alleles (72.8%) than do people who are A3B II or ID (67%) (P = 0.015) (Table 2). Interestingly, the frequency of the ACE Alu insertion alleles was also higher among people who carry the higher A3B deletion alleles in geographically different locations (Fig. 1). The cause for this association between the A3B deletion and ACE Alu insertion is not clear. However, there are several possible explanations: (1) The ACE Alu insertion and the A3B deletion occurred as independent events. Selection pressure resulted in a non-random distribution of these two genetic alterations. (2) The two I/D polymorphisms have interactive effects on the survival of humanity. (3) Cause-and-effect relationship exists between A3B deletion and ACE Alu insertion. The selection pressure for the existence of the A3B deletion is unknown. DNA mutations induced by elevated expression of A3B have been reported in liver cancer and breast cancer [36], [37]. Thus, the A3B deletion may offer a reduced risk for the development of certain cancers. Links between the lack of the ACE Alu insertion and hypertension, as well as other diseases, have been reported [2]–[7]. It is therefore also possible that A3B deletion is detrimental to human survival, but this outcome could be partially offset by the ACE Alu insertion, leading to the apparent association of these two genetic alterations.

It is intriguing to consider that the A3B deletion may be the cause of the ACE Alu insertion. Members of the APOBEC3 (A3) family were initially discovered as host restriction factors for exogenous retroviruses [13], [14]. Their roles in controlling the retrotransposition of endogenous retroelements have also attracted much attention in recent years [38], [39]. The sequence variability of primate A3 genes indicates that this gene cluster has been subjected to strong positive selective pressure for more than 30 million years, clearly preceding the appearance of primate lentiviruses [26]. By contrast, there is a striking evolutionary coincidence between the expansion of the A3 gene cluster and the abrupt drop in retrotransposon activity that took place in primates 35–50 million years ago [40]. Moreover, A3 proteins are expressed at relatively high levels in germ cells and embryonic stem cells, where retrotransposition occurs actively and can be inherited by the offspring [16], [20]. A3B is a powerful inhibitor of Alu retrotransposition. It is the only member of the A3 family that is consistently localized to the nucleus, where Alu is reverse-transcribed and inserted into other loci [17]. Endogenous A3B has been detected in embryonic stem cells, where it modulates LINE-1 retrotransposition [16]. If the A3B deletion is indeed the cause of the ACE Alu insertion, then the occurrence of the A3B deletion has great significance for the history of modern humanity, because the ACE Alu insertion is believed to have greatly promoted human adaptability and human survival rates during the out-of-Africa expansion [8]. It should be noted that the A3B deletion may have enabled more than one Alu insertion event, so its geographic distribution may also have been influenced by other Alu I/D polymorphisms. In particular, some other Alu I/D polymorphisms present geographic distributions analogous to that of the ACE I/D polymorphism (Table S1) [28]. This is the first report of an association between A3B deletion and Alu insertion. Further study is required to establish what selection pressures may have contributed to this association.

## Supporting Information

File S1 **Calculation for the geographic distributions of A3B deletion, ACE Alu insertion and other three Alu insertions.**
(XLS)

Table S1 **Geographic distributions of A3B deletion, ACE Alu insertion and other three Alu insertions.**
(DOC)

Table S2 **A3B genotype/allele distributions in subjects stratified by sex.**
(DOC)

Table S3 **Characteristics of male subjects grouped by ACE and A3B genotypes.**
(DOC)

Table S4 **Characteristics of female subjects grouped by ACE and A3B genotypes.**
(DOC)

Table S5 **Characteristics of male subjects grouped by ACE genotypes.**
(DOC)

Table S6 **Characteristics of female subjects grouped by ACE genotypes.**
(DOC)

## References

[1] Rigat B, Hubert C, Alhenc-Gelas F, Cambien F, Corvol P, et al (1990) An insertion/deletion polymorphism in the angiotensin I-converting enzyme gene accounting for half the variance of serum enzyme levels. J Clin Invest 86: 1343–1346. 1976655 10.1172/JCI114844 PMC296868

[2] Di Pasquale P, Cannizzaro S, Paterna S (2004) Does angiotensin-converting enzyme gene polymorphism affect blood pressure? Findings after 6 years of follow-up in healthy subjects. Eur J Heart Fail 6: 11–16. 15012913 10.1016/j.ejheart.2003.07.009

[3] Feng Y, Niu T, Xu X, Chen C, Li Q, et al (2002) Insertion/deletion polymorphism of the ACE gene is associated with type 2 diabetes. Diabetes 51: 1986–1988. 12031990 10.2337/diabetes.51.6.1986

[4] Schunkert H, Hense HW, Holmer SR, Stender M, Perz S, et al (1994) Association between a deletion polymorphism of the angiotensin-converting-enzyme gene and left ventricular hypertrophy. N Engl J Med 330: 1634–1638. 8177269 10.1056/NEJM199406093302302

[5] Harden PN, Geddes C, Rowe PA, McIlroy JH, Boulton-Jones M, et al (1995) Polymorphisms in angiotensin-converting-enzyme gene and progression of IgA nephropathy. Lancet 345: 1540–1542. 7791440 10.1016/s0140-6736(95)91088-3

[6] Medeiros R, Vasconcelos A, Costa S, Pinto D, Lobo F, et al (2004) Linkage of angiotensin I-converting enzyme gene insertion/deletion polymorphism to the progression of human prostate cancer. J Pathol 202: 330–335. 14991898 10.1002/path.1529

[7] Eisenmann JC, Sarzynski MA, Glenn K, Rothschild M, Heelan KA (2009) ACE I/D genotype, adiposity, and blood pressure in children. Cardiovasc Diabetol 8: 14. 19291311 10.1186/1475-2840-8-14 PMC2658665

[8] Li X, Sun X, Jin L, Xue F (2011) Worldwide spatial genetic structure of angiotensin-converting enzyme gene: a new evolutionary ecological evidence for the thrifty genotype hypothesis. Eur J Hum Genet 19: 1002–1008. 21559052 10.1038/ejhg.2011.66 PMC3179366

[9] Ehlers MR, Riordan JF (1989) Angiotensin-converting enzyme: new concepts concerning its biological role. Biochemistry 28: 5311–5318. 2476171 10.1021/bi00439a001

[10] Lieberman J, Sastre A (1983) Angiotensin-converting enzyme activity in postmortem human tissues. Lab Invest 48: 711–717. 6304422

[11] Hubert C, Houot AM, Corvol P, Soubrier F (1991) Structure of the angiotensin I-converting enzyme gene. Two alternate promoters correspond to evolutionary steps of a duplicated gene. J Biol Chem 266: 15377–15383. 1651327

[12] Jarmuz A, Chester A, Bayliss J, Gisbourne J, Dunham I, et al (2002) An anthropoid-specific locus of orphan C to U RNA-editing enzymes on chromosome 22. Genomics 79: 285–296. 11863358 10.1006/geno.2002.6718

[13] Bishop KN, Holmes RK, Sheehy AM, Davidson NO, Cho SJ, et al (2004) Cytidine deamination of retroviral DNA by diverse APOBEC proteins. Curr Biol 14: 1392–1396. 15296758 10.1016/j.cub.2004.06.057

[14] Dang Y, Wang X, Esselman WJ, Zheng YH (2006) Identification of APOBEC3DE as another antiretroviral factor from the human APOBEC family. J Virol 80: 10522–10533. 16920826 10.1128/JVI.01123-06 PMC1641744

[15] Stenglein MD, Harris RS (2006) APOBEC3B and APOBEC3F inhibit L1 retrotransposition by a DNA deamination-independent mechanism. J Biol Chem 281: 16837–16841. 16648136 10.1074/jbc.M602367200

[16] Wissing S, Montano M, Garcia-Perez JL, Moran JV, Greene WC (2011) Endogenous APOBEC3B restricts LINE-1 retrotransposition in transformed cells and human embryonic stem cells. J Biol Chem 286: 36427–36437. 21878639 10.1074/jbc.M111.251058 PMC3196128

[17] Bogerd HP, Wiegand HL, Hulme AE, Garcia-Perez JL, O'Shea KS, et al (2006) Cellular inhibitors of long interspersed element 1 and Alu retrotransposition. Proc Natl Acad Sci U S A 103: 8780–8785. 16728505 10.1073/pnas.0603313103 PMC1482655

[18] Tan L, Sarkis PT, Wang T, Tian C, Yu XF (2009) Sole copy of Z2-type human cytidine deaminase APOBEC3H has inhibitory activity against retrotransposons and HIV-1. FASEB J 23: 279–287. 18827027 10.1096/fj.07-088781 PMC2626612

[19] Zhang W, Zhang X, Tian C, Wang T, Sarkis PT, et al (2008) Cytidine deaminase APOBEC3B interacts with heterogeneous nuclear ribonucleoprotein K and suppresses hepatitis B virus expression. Cell Microbiol 10: 112–121. 17672864 10.1111/j.1462-5822.2007.01020.x

[20] Refsland EW, Stenglein MD, Shindo K, Albin JS, Brown WL, et al (2010) Quantitative profiling of the full APOBEC3 mRNA repertoire in lymphocytes and tissues: implications for HIV-1 restriction. Nucleic Acids Res 38: 4274–4284. 20308164 10.1093/nar/gkq174 PMC2910054

[21] Kidd JM, Newman TL, Tuzun E, Kaul R, Eichler EE (2007) Population stratification of a common APOBEC gene deletion polymorphism. PLoS Genet 3: e63. 17447845 10.1371/journal.pgen.0030063 PMC1853121

[22] Zhang T, Cai J, Chang J, Yu D, Wu C, et al (2012) Evidence of associations of APOBEC3B gene deletion with susceptibility to persistent HBV infection and hepatocellular carcinoma. Hum Mol Genet 10.1093/hmg/dds513 23213177

[23] Jha P, Sinha S, Kanchan K, Qidwai T, Narang A, et al (2012) Deletion of the APOBEC3B gene strongly impacts susceptibility to falciparum malaria. Infect Genet Evol 12: 142–148. 22108670 10.1016/j.meegid.2011.11.001

[24] Conticello SG, Thomas CJ, Petersen-Mahrt SK, Neuberger MS (2005) Evolution of the AID/APOBEC family of polynucleotide (deoxy)cytidine deaminases. Mol Biol Evol 22: 367–377. 15496550 10.1093/molbev/msi026

[25] Zhang J, Webb DM (2004) Rapid evolution of primate antiviral enzyme APOBEC3G. Hum Mol Genet 13: 1785–1791. 15198990 10.1093/hmg/ddh183

[26] Sawyer SL, Emerman M, Malik HS (2004) Ancient adaptive evolution of the primate antiviral DNA-editing enzyme APOBEC3G. PLoS Biol 2: E275. 15269786 10.1371/journal.pbio.0020275 PMC479043

[27] Ismail M, Akhtar N, Nasir M, Firasat S, Ayub Q, et al (2004) Association between the angiotensin-converting enzyme gene insertion/deletion polymorphism and essential hypertension in young Pakistani patients. J Biochem Mol Biol 37: 552–555. 15479618 10.5483/bmbrep.2004.37.5.552

[28] Stoneking M, Fontius JJ, Clifford SL, Soodyall H, Arcot SS, et al (1997) Alu insertion polymorphisms and human evolution: evidence for a larger population size in Africa. Genome Res 7: 1061–1071. 9371742 10.1101/gr.7.11.1061 PMC310683

[29] Vargas-Alarcon G, Hernandez-Pacheco G, Rodriguez-Perez JM, Perez-Hernandez N, Pavon Z, et al (2003) Angiotensin-converting enzyme gene (ACE) insertion/deletion polymorphism in Mexican populations. Hum Biol 75: 889–896. 15018037 10.1353/hub.2004.0012

[30] Rupert JL, Kidd KK, Norman LE, Monsalve MV, Hochachka PW, et al (2003) Genetic polymorphisms in the Renin-Angiotensin system in high-altitude and low-altitude Native American populations. Ann Hum Genet 67: 17–25. 12556231 10.1046/j.1469-1809.2003.00004.x

[31] Herrmann FH, Salazar-Sanchez L, Schuster G, Jimenez-Arce G, Grimm R, et al (2004) Prevalence of eight molecular markers associated with thrombotic diseases in six Amerindian tribes and two African groups of Costa Rica. Am J Hum Biol 16: 82–86. 14689519 10.1002/ajhb.10229

[32] Crews DE, Fitton LJ, Kottke BA, Kamboh MI (2004) Population genetics of apolipoproteins A-IV, E, and H, and the angiotensin converting enzyme (ACE): associations with lipids, and apolipoprotein levels in American Samoans. Am J Phys Anthropol 124: 364–372. 15252864 10.1002/ajpa.10355

[33] Rigat B, Hubert C, Corvol P, Soubrier F (1992) PCR detection of the insertion/deletion polymorphism of the human angiotensin converting enzyme gene (DCP1) (dipeptidyl carboxypeptidase 1). Nucleic Acids Res 20: 1433. 10.1093/nar/20.6.1433-a PMC312213 1313972

[34] Shanmugam V, Sell KW, Saha BK (1993) Mistyping ACE heterozygotes. PCR Methods Appl 3: 120–121. 8268786 10.1101/gr.3.2.120

[35] Lindpaintner K, Pfeffer MA, Kreutz R, Stampfer MJ, Grodstein F, et al (1995) A prospective evaluation of an angiotensin-converting-enzyme gene polymorphism and the risk of ischemic heart disease. N Engl J Med 332: 706–711. 7854377 10.1056/NEJM199503163321103

[36] Xu R, Zhang X, Zhang W, Fang Y, Zheng S, et al (2007) Association of human APOBEC3 cytidine deaminases with the generation of hepatitis virus B x antigen mutants and hepatocellular carcinoma. Hepatology 46: 1810–1820. 17847074 10.1002/hep.21893

[37] Burns MB, Lackey L, Carpenter MA, Rathore A, Land AM, et al (2013) APOBEC3B is an enzymatic source of mutation in breast cancer. Nature 494: 366–370. 23389445 10.1038/nature11881 PMC3907282

[38] Chiu YL, Greene WC (2008) The APOBEC3 cytidine deaminases: an innate defensive network opposing exogenous retroviruses and endogenous retroelements. Annu Rev Immunol 26: 317–353. 18304004 10.1146/annurev.immunol.26.021607.090350

[39] Arias JF, Koyama T, Kinomoto M, Tokunaga K (2012) Retroelements versus APOBEC3 family members: No great escape from the magnificent seven. Front Microbiol 3: 275. 22912627 10.3389/fmicb.2012.00275 PMC3418512

[40] Mouse Genome Sequencing C, Waterston RH, Lindblad-Toh K, Birney E, Rogers J, et al (2002) Initial sequencing and comparative analysis of the mouse genome. Nature 420: 520–562. 12466850 10.1038/nature01262

