# Supplementary material for: Characterization of the Relationship between APOBEC3B Deletion and ACE Alu Insertion
Source: PLoS One. 2013 May 24;8(5):e64809. doi: 10.1371/journal.pone.0064809 (PMC3663847; doi:10.1371/journal.pone.0064809)
Supplement: Table S1 — Geographic distributions of A3B deletion, ACE Alu insertion and other three Alu insertions. (DOC) [file pone.0064809.s002.doc]

**Table S1** Geographic distributions of A3B deletion, ACE Alu insertion and other three Alu insertions

| Geographic Region | A3B deletion | Alu insertion frequency (%) | | | |
| --- | --- | --- | --- | --- | --- |
|  |  | ACE Alu | FXIIIB | PV92 | TPA25 |
| Africa | 0.9 | 34.1 | 9.7 | 23.8 | 26.2 |
| Middle East | 7.7 | 32.6 | 39.0 | 30.0 | 44.0 |
| Europe | 6.5 | 46.7 | 46.2 | 25.6 | 53.6 |
| C/S Asia ^a^ | 17.8 | 55.7 | 61.3 | 41.5 | 49.0 |
| East Asia | 36.9 | 63.3 | 78.3 | 82.0 | 53.0 |
| America ^b^ | 47.7 | 73.8 | 85.0 | 64.8 | 44.3 |
| Oceania ^c^ | 92.9 | 83.5 | 83.7 | 67.7 | 44.3 |

^a^ ‘C/S Asia’ corresponds to Central South Asia. ^b^ refers to Amerindians; ^c^ refers to Oceania aboriginals.
