# Supplementary material for: Characterization of the Relationship between APOBEC3B Deletion and ACE Alu Insertion
Source: PLoS One. 2013 May 24;8(5):e64809. doi: 10.1371/journal.pone.0064809 (PMC3663847; doi:10.1371/journal.pone.0064809)
Supplement: Table S2 — A3B genotype/allele distributions in subjects stratified by sex. (DOC) [file pone.0064809.s003.doc]

**Table S2** A3B genotype/allele distributions in subjects stratified by sex

| Grouped by sex | Genotype counts | | | HWE  P-value | Allele frequency | | P-value |
| --- | --- | --- | --- | --- | --- | --- | --- |
|  | II | ID | DD |  | I | D |  |
| males | 573 | 742 | 124 | 0.001 | 0.656 | 0.344 | 0.888 ^a^ |
| females | 521 | 706 | 111 | < 0.001 | 0.653 | 0.347 |  |

^a^ Comparison between the allele frequencies in males and in females with Chi-square test.
