# Supplementary material for: Characterization of the Relationship between APOBEC3B Deletion and ACE Alu Insertion
Source: PLoS One. 2013 May 24;8(5):e64809. doi: 10.1371/journal.pone.0064809 (PMC3663847; doi:10.1371/journal.pone.0064809)
Supplement: Table S3 — Characteristics of male subjects grouped by ACE and A3B genotypes. (DOC) [file pone.0064809.s004.doc]

**Table S3** Characteristics of male subjects grouped by ACE and A3B genotypes

| Characteristic | Mean ± SD (No. of subjects measured) | | P-value |
| --- | --- | --- | --- |
|  | ACE D/A3B D carriers | others |  |
| Age (years) | 40.8 ± 12.1 (235) | 41.6 ± 12.1 (448) | 0.318 |
| BMI (kg/m^2^) | 26.0 ± 3.3 (205) | 26.0 ± 3.2 (388) | 0.963 |
| Heart rate (beats/min) | 77.6 ± 11.4 (211) | 75.9 ± 10.2 (399) | 0.160 |
| Blood pressure (mm Hg) |  |  |  |
| Systolic | 135.5 ± 18.6 (211) | 134.4 ± 18.1 (399) | 0.518 |
| Diastolic | 83.8 ± 12.1 (211) | 83.3 ± 11.4 (399) | 0.785 |
| Plasma glucose (mmol/L) | 5.86 ± 2.03 (213) | 5.60 ± 1.26 (403) | 0.327 |
| Serum lipid (mmol/L) |  |  |  |
| Total cholesterol | 4.87 ± 0.86 (214) | 4.88 ± 0.92 (403) | 0.929 |
| Triglycerides | 1.90 ± 1.36 (214) | 1.85 ± 1.40 (403) | 0.543 |
| HDL-cholesterol | 1.55 ± 0.51 (172) | 1.54 ± 0.58 (342) | 0.461 |
| LDL-cholesterol | 3.11 ± 0.81 (172) | 3.06 ± 0.78 (342) | 0.568 |
| HDL-C/LDL-C ratio | 0.53 ± 0.20 (172) | 0.53 ± 0.23 (342) | 0.903 |
| Renal function indexes |  |  |  |
| BUN (mmol/L) | 5.25 ± 1.29 (212) | 5.38 ± 1.34 (407) | 0.250 |
| Urinary protein | —— ^a^ (220) | —— ^a^ (427) | 0.417 |
| Urinary occult blood | —— ^a^ (220) | —— ^a^ (427) | 0.962 |
| Liver function indexes (U/L) |  |  |  |
| ALT | 33.2 ± 23.7 (225) | 31.3 ± 21.4 (430) | 0.563 |
| γ–GT | 45.3 ± 40.4 (225) | 43.8 ± 46.5 (430) | 0.444 |
| AST | 26.4 ± 12.4 (226) | 24.8 ± 10.0 (430) | 0.409 |

^a^ belong to categorical variables.

Abbreviations: BMI, body mass index; HDL, high density lipoprotein; LDL, low density lipoprotein; BUN, blood urea nitrogen; ALT, alanine aminotransferase; γ–GT, gamma-glutamyl transpeptidase; AST, aspartate aminotransferase
