# Supplementary material for: Characterization of the Relationship between APOBEC3B Deletion and ACE Alu Insertion
Source: PLoS One. 2013 May 24;8(5):e64809. doi: 10.1371/journal.pone.0064809 (PMC3663847; doi:10.1371/journal.pone.0064809)
Supplement: Table S4 — Characteristics of female subjects grouped by ACE and A3B genotypes. (DOC) [file pone.0064809.s005.doc]

**Table S4** Characteristics of female subjects grouped by ACE and A3B genotypes

| Characteristic | Mean ± SD (No. of subjects measured) | | P-value |
| --- | --- | --- | --- |
|  | ACE D/A3B D carriers | others |  |
| Age (years) | 42.4 ± 12.5 (174) | 41.7 ± 12.5 (342) | 0.573 |
| BMI (kg/m^2^) | 23.6 ± 3.4 (153) | 23.3 ± 3.2 (295) | 0.343 |
| Heart rate (beats/min) | 80.8 ± 11.3 (161) | 79.3 ± 11.1 (312) | 0.114 |
| Blood pressure (mm Hg) |  |  |  |
| Systolic | 126.5 ± 20.4 (161) | 125.6 ± 19.3 (312) | 0.475 |
| Diastolic | 76.5 ± 11.4 (161) | 75.6 ± 12.3 (312) | 0.264 |
| Plasma glucose (mmol/L) | 5.23 ± 0.78 (158) | 5.26 ± 0.76 (313) | 0.821 |
| Serum lipid (mmol/L) |  |  |  |
| Total cholesterol | 4.86 ± 0.89 (155) | 4.78 ± 0.91 (312) | 0.309 |
| Triglycerides | 1.23 ± 0.76 (155) | 1.26 ± 0.94 (312) | 0.663 |
| HDL-cholesterol | 1.90 ± 0.66 (119) | 1.96 ± 0.76 (258) | 0.783 |
| LDL-cholesterol | 2.96 ± 0.79 (119) | 2.84 ± 0.76 (258) | 0.158 |
| HDL-C/LDL-C ratio | 0.69 ± 0.31 (119) | 0.75 ± 0.41 (258) | 0.403 |
| Renal function indexes |  |  |  |
| BUN (mmol/L) | 4.52 ± 1.18 (155) | 4.61 ± 1.20 (311) | 0.454 |
| Urinary protein | —— ^a^ (154) | —— ^a^ (314) | 0.976 |
| Urinary occult blood | —— ^a^ (154) | —— ^a^ (314) | 0.565 |
| Liver function indexes (U/L) |  |  |  |
| ALT | 20.0 ± 23.4 (159) | 20.9 ± 29.2 (327) | 0.784 |
| γ–GT | 21.5 ± 17.2 (159) | 21.7 ± 20.2 (327) | 0.887 |
| AST | 22.8 ± 19.3 (159) | 21.3 ± 10.5 (329) | 0.484 |

^a^ belong to categorical variables.

Abbreviations: BMI, body mass index; HDL, high density lipoprotein; LDL, low density lipoprotein; BUN, blood urea nitrogen; ALT, alanine aminotransferase; γ–GT, gamma-glutamyl transpeptidase; AST, aspartate aminotransferase.
