# Supplementary material for: Characterization of the Relationship between APOBEC3B Deletion and ACE Alu Insertion
Source: PLoS One. 2013 May 24;8(5):e64809. doi: 10.1371/journal.pone.0064809 (PMC3663847; doi:10.1371/journal.pone.0064809)
Supplement: Table S5 — Characteristics of male subjects grouped by ACE genotypes. (DOC) [file pone.0064809.s006.doc]

**Table S5** Characteristics of male subjects grouped by ACE genotypes

| Characteristic | Mean ± SD (No. of subjects measured) | | | P-value |
| --- | --- | --- | --- | --- |
|  | II | ID | DD |  |
| Age (years) | 41.4 ± 12.3 (313) | 41.3 ± 11.9 (297) | 41.0 ± 12.5 (73) | 0.936 |
| BMI (kg/m^2^) | 25.9 ± 3.4 (271) | 26.1 ± 3.2 (262) | 26.1 ± 3.4 (60) | 0.765 |
| Heart rate (beats/min) | 76.1 ± 10.3 (278) | 76.9 ± 11.2 (268) | 76.3 ± 9.6 (64) | 0.829 |
| Blood pressure (mm Hg) |  |  |  |  |
| Systolic | 133.6 ± 17.9 (278) | 135.3 ± 18.6 (268) | 138.1 ± 18.7 (64) | 0.173 |
| Diastolic | 83.1 ± 11.5 (278) | 83.9 ± 11.9 (268) | 83.4 ± 11.2 (64) | 0.724 |
| Plasma glucose (mmol/L) | 5.59 ± 1.15 (285) | 5.71 ± 1.58 (263) | 6.04 ± 2.65 (68) | 0.928 |
| Serum lipid (mmol/L) |  |  |  |  |
| Total cholesterol | 4.86 ± 0.94 (286) | 4.90 ± 0.87 (264) | 4.83 ± 0.84 (67) | 0.708 |
| Triglycerides | 1.82 ± 1.39 (286) | 1.90 ± 1.36 (264) | 1.95 ± 1.51 (67) | 0.468 |
| HDL-cholesterol | 1.56 ± 0.59 (239) | 1.52 ± 0.53 (219) | 1.57 ± 0.50 (56) | 0.643 |
| LDL-cholesterol | 3.08 ± 0.77 (239) | 3.09 ± 0.82 (219) | 3.00 ± 0.71 (56) | 0.784 |
| HDL-C/LDL-C ratio | 0.53 ± 0.23 (239) | 0.52 ± 0.21 (219) | 0.54 ± 0.20 (56) | 0.516 |
| Renal function indexes |  |  |  |  |
| BUN (mmol/L) | 5.44 ± 1.32 (288) | 5.19 ± 1.26 (264) | 5.50 ± 1.51 (67) | **0.044** |
| Urinary protein | —— ^a^ (300) | —— ^a^ (280) | —— ^a^ (67) | 0.606 |
| Urinary occult blood | —— ^a^ (300) | —— ^a^ (280) | —— ^a^ (67) | 0.312 |
| Liver function indexes (U/L) |  |  |  |  |
| ALT | 31.3 ± 19.3 (303) | 31.6 ± 21.9 (282) | 36.0 ± 32.9 (70) | 0.831 |
| γ–GT | 43.7 ± 44.1 (303) | 45.2 ± 45.9 (282) | 43.3 ± 40.4 (70) | 0.752 |
| AST | 24.7 ± 9.2 (303) | 25.8 ± 12.0 (283) | 26.3 ± 13.1 (70) | 0.951 |
| BUN (mmol/L) | 5.41 ± 1.31 (265) | 5.16 ± 1.24 (249) | 5.56 ± 1.54 (59) | **0.031** ^b^ |

^a^ belong to categorical variables.

^b^ p-value from analysis of covariance including subjects whose BMI data were available.

Abbreviations: BMI, body mass index; HDL, high density lipoprotein; LDL, low density lipoprotein; BUN, blood urea nitrogen; ALT, alanine aminotransferase; γ–GT, gamma-glutamyl transpeptidase; AST, aspartate aminotransferase.
