# Supplementary material for: Characterization of the Relationship between APOBEC3B Deletion and ACE Alu Insertion
Source: PLoS One. 2013 May 24;8(5):e64809. doi: 10.1371/journal.pone.0064809 (PMC3663847; doi:10.1371/journal.pone.0064809)
Supplement: Table S6 — Characteristics of female subjects grouped by ACE genotypes. (DOC) [file pone.0064809.s007.doc]

**Table S6** Characteristics of female subjects grouped by ACE genotypes

| Characteristic | Mean ± SD (No. of subjects measured) | | | P-value |
| --- | --- | --- | --- | --- |
|  | II | ID | DD |  |
| Age (years) | 41.7 ± 12.5 (229) | 42.6 ± 12.9 (236) | 40.3 ± 10.4 (51) | 0.603 |
| BMI (kg/m^2^) | 23.4 ± 3.1 (199) | 23.6 ± 3.5 (204) | 23.2 ± 2.8 (45) | 0.774 |
| Heart rate (beats/min) | 79.5 ± 10.7 (209) | 79.6 ± 11.2 (218) | 82.5 ± 12.7 (46) | 0.294 |
| Blood pressure (mm Hg) |  |  |  |  |
| Systolic | 125.9 ± 19.4 (209) | 126.0 ± 19.9 (218) | 125.1 ± 20.1 (46) | 0.948 |
| Diastolic | 76.1 ± 12.6 (209) | 75.5 ± 15.5 (218) | 76.6 ± 11.8 (46) | 0.853 |
| Plasma glucose (mmol/L) | 5.26 ± 0.83 (211) | 5.25 ± 0.74 (212) | 5.25 ± 0.60 (48) | 0.540 |
| Serum lipid (mmol/L) |  |  |  |  |
| Total cholesterol | 4.79 ± 0.92 (207) | 4.83 ± 0.92 (214) | 4.77 ± 0.75 (46) | 0.905 |
| Triglycerides | 1.25 ± 0.93 (207) | 1.28 ± 0.88 (214) | 1.17 ± 0.65 (46) | 0.904 |
| HDL-cholesterol | 1.97 ± 0.77 (171) | 1.92 ± 0.68 (171) | 1.85 ± 0.74 (35) | 0.633 |
| LDL-cholesterol | 2.84 ± 0.76 (171) | 2.89 ± 0.77 (171) | 3.03 ± 0.73 (35) | 0.349 |
| HDL-C/LDL-C ratio | 0.76 ± 0.41 (171) | 0.72 ± 0.36 (171) | 0.65 ± 0.32 (35) | 0.280 |
| Renal function indexes |  |  |  |  |
| BUN (mmol/L) | 4.55 ± 1.09 (209) | 4.63 ± 1.34 (212) | 4.47 ± 0.94 (45) | 0.901 |
| Urinary protein | —— ^a^ (212) | —— ^a^ (212) | —— ^a^ (44) | 0.311 |
| Urinary occult blood | —— ^a^ (212) | —— ^a^ (212) | —— ^a^ (44) | 0.616 |
| Liver function indexes (U/L) |  |  |  |  |
| ALT | 19.5 ± 16.0 (219) | 22.2 ± 37.1 (221) | 18.5 ± 10.5 (46) | 0.697 |
| γ-GT | 22.0 ± 20.1 (219) | 20.8 ± 17.5 (221) | 23.8 ± 23.2 (46) | 0.872 |
| AST | 21.0 ± 10.0 (221) | 22.9 ± 17.9 (221) | 20.3 ± 6.5 (46) | 0.339 |

^a^ belong to categorical variables.

Abbreviations: BMI, body mass index; HDL, high density lipoprotein; LDL, low density lipoprotein; BUN, blood urea nitrogen; ALT, alanine aminotransferase; γ–GT, gamma-glutamyl transpeptidase; AST, aspartate aminotransferase.
